# The Effectiveness of a Primary Care Diabetes Education and Self‐Management Program in Ireland: A 6‐Month Follow‐Up Study

**DOI:** 10.1002/edm2.70036

**Published:** 2025-02-19

**Authors:** Clodagh Scannell, Tonya O'Neill, Anne Griffin

**Affiliations:** ^1^ School of Allied Health University of Limerick Limerick Ireland; ^2^ Nutrition & Oncology Research Group, School of Food & Nutritional Sciences University College Cork Cork Ireland; ^3^ HSE Mid‐West Health Region Barack View Primary Care Health Centre Limerick Ireland; ^4^ School of Allied Health, Faculty of Education and Science University of Limerick Limerick Ireland; ^5^ Health Research Institute University of Limerick Limerick Ireland

**Keywords:** DESMOND, interventions, self‐management, structured education, type 2 diabetes

## Abstract

**Aims:**

Self‐management education is recognised as an essential element of comprehensive diabetes care. This study aims to assess the impact of the DESMOND (Diabetes Education and Self‐Management for Ongoing and Newly Diagnosed) structured diabetes self‐management education programme administered by a registered dietitian in a primary‐care setting on key clinical indicators (HbA1c, weight and BMI) in participants who returned for the locally developed 6‐month follow‐up session.

**Methods:**

A retrospective analysis was conducted of participants who attended the DESMOND 6‐h structured diabetes self‐management education programme and returned for the locally developed follow‐up programme during 2018 in the Midwest of Ireland. Paired sample t‐tests and McNemar chi‐square tests were used to assess any differences between baseline and 6 months post‐intervention.

**Results:**

There were 66 participants, mean age of 63 years. At follow‐up, HbA1c was reduced by 6.45 mmol/mol (standard deviation (SD): 15.02 mmol/mol, *p* = 0.006). The number of participants below the 53 mmol/mol cut‐off increased from 52% at baseline to 71% at follow‐up (*p* < 0.001). A mean weight reduction of 1.4 kg (SD: 4.4 kg, *p* = 0.21) was found at follow‐up. Those in the overweight BMI category decreased from 30.2% to 26.4%, a clinically significant result.

**Conclusion:**

Better glycaemic control and clinically significant improvements in BMI and weight were seen at 6 months among participants who attended the DESMOND program and returned for the locally developed follow‐up session. This supports the emerging evidence of the effectiveness of self‐management education for diabetes care. Further research is required to determine the optimal contact time and frequency of sessions required in order to sustain the observed improvement in clinical outcomes.

## Graphical abstract

Our study found that participants who completed the DESMOND diabetes self‐management programme showed significant improvements in HbA1c, weight, and BMI at 6 months, supporting the effectiveness of self‐management education in diabetes care delivered by dietitians in primary care.

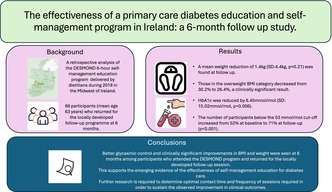

**Summary**

- Type 2 diabetes mellitus has long been acknowledged as a significant challenge for individuals, healthcare professionals, and healthcare systems. Diabetes self‐management programmes (DSME) are proposed as effective interventions to address these challenges.
- Our findings revealed a significant reduction in HbA1c levels and a notable impact on BMI among adults with type 2 diabetes mellitus who attended a DSME programme delivered in primary care. Notably, over 90% of participants were overweight or obese, highlighting the relevance of weight management in diabetes care.
- Amidst rising diabetes rates, this research underscores the imperative for enhancing DSME uptake and attendance, providing compelling evidence for future service models and healthcare policies.

## 1 Introduction

Type 2 diabetes mellitus imposes a substantial burden on individuals, healthcare professionals (HCPs), and the broader health system [1]. People living with type 2 diabetes mellitus face daily decisions affecting their health, including dietary choices, medication compliance, and physical activity. These decisions are often made with limited guidance from health care professionals [2]. Structured diabetes self‐management education (DSME) is now integral to optimal diabetes care, alongside glucose‐lowering medications and psychological support [2, 3]. These programmes aim to improve participants knowledge of how to incorporate health‐promoting behaviours into their daily lives. This includes how to improve metabolic control, decisions around medication, and improve quality of life [2, 3]. In addition, group education is cost‐effective [4] and provides opportunities for shared experiences [2]. The Institute of Public Health in Ireland have reported that the increasing prevalence of type 2 diabetes mellitus is driven primarily by the ageing population [5]. The reported prevalence of diagnosed and undiagnosed type 2 diabetes mellitus is 8.4% in those over 50 years, with a higher prevalence in men (10.3%) than women (6.6%) [6, 7]. Prevalence also increases with advancing age [6, 7]. Among individuals in this age group, 26% reported microvascular complications, and 15% reported macrovascular complications [7]. The American Diabetes Association “Standards of Care in Diabetes” advises on therapeutic goals of clinical parameters for glycaemic and weight management control in order to reduce or delay the micro and macrovascular complications of diabetes [8, 9]. Diabetes also exerts a significant financial strain on the Irish health service, costing approximately 10% (€1.7 billion) of the healthcare budget [10]. This thereby supports the need for early initiation of DSME.

Given the average Irish life expectancy of 82 years, there is a critical need to centre the ‘end‐to‐end’ treatment of diabetes within the primary care setting [11]. There is an onus on HCPs to help people to understand that DSME should be included as part of their treatment plan in line with the new emerging models of integrated care [11]. The current service model of integrated care for type 2 diabetes mellitus in Ireland advocates for structured patient education to empower individuals, improve quality of life, and promote active self‐management [2, 11] Referrals to DSME is ideally within 3 months of diagnosis [2, 11]. Quality standards for the provision of DSME have been established [11, 12]. Currently, there are three structured education programmes for the management of type 2 diabetes mellitus [11]. These include DISCOVER Diabetes ‐ Type 2 (Diabetes Insights and Self Care Options via Education and Reflection), CODE (Community Orientated Diabetes Education) and DESMOND (Diabetes Education and Self‐Management for Ongoing and Newly Diagnosed) [11], with DESMOND currently being the only programme to be validated using a randomised controlled trial [13].

However, the impact of DSME on clinical markers within Irish services is inadequately published to date, creating a knowledge gap [14]. Documenting participant outcomes is important to establish the efficacy of DSME interventions and service modelling. This study aims to evaluate the effectiveness of the DESMOND structured education programme in people with newly diagnosed and existing diabetes who attended a locally developed follow‐up session 6 months post‐DESMOND in a primary care centre in the Midwest of Ireland.

## 2 Methods

### 2.1 Study Design

A retrospective chart review was used to extract patient outcome data from the dietetic records of participants who completed DSME during 2018 in the Health Service Executive Midwest Community Healthcare area. Ethical approval was granted by the HSE University Hospital Limerick Research Ethics Committee.

### 2.2 Population and DSME Intervention

Diabetes Education and Self‐Management for Ongoing and Newly Diagnosed (DESMOND) is an evidence‐based group education program for people with type 2 diabetes mellitus, focusing on long‐term self‐management through behaviour change [15]. Programme completion requires attendance at 6‐h of education either on one whole day or two half days in groups of up to 12 people with type 2 diabetes mellitus who can bring an accompanying person [11, 16]. In the HSE Midwest service, local policy is to offer a follow‐up session after 6‐months. The combination of DESMOND group and the 6‐month locally developed group will hereafter be called ‘The Midwest DSME’.

Data was collected from one primary care centre in the Midwest of Ireland. Baseline data were collected from participants prior to attending the first session and again at the 6‐month follow‐up. Participants were eligible for inclusion in this study if they attended the Midwest DSME during 2018. The exclusion criteria were those who did not attend any or only one session. Attendance at both sessions was deemed necessary in order to obtain the relevant clinical measurements (weight and BMI) and evaluate change 6 months post DESMOND.

### 2.3 Clinical Outcome Measures

The primary outcome was glycated haemoglobin (HbA1c) levels. HbA1c reflects average plasma glucose over the previous 8–12 weeks and is the preferred test for assessing glycaemic control. HbA1c values are provided per routine collection of the clinical parameters among participants attending DESMOND, and no additional blood samples were required. Clinical goals used for HbA1c were < 53 mmol/mol (< 7%) for adults and older adults who are otherwise healthy, with few coexisting illnesses and intact cognitive function and functional status [17]. Weight (kg) and BMI (kg/m^2^) data were measured by a registered dietitian and collected onsite. The goal for weight loss was set at 3%–7% of baseline weight, which is shown to improve glycaemia and other cardiovascular risk factors [9].

### 2.4 Statistical Analysis

A descriptive data analysis was conducted using SPSS version 28 [18]. Continuous variables were summarised using means, standard deviations, and ranges. Categorical variables were summarised using counts and percentages. Shapiro–Wilk tests were used to test the normality of the data. BMI was categorised according to the WHO classification (underweight, normal weight, overweight and obese) [19]. All missing data was assumed to be randomly missing and were not replaced. Paired sample *t*‐tests were used to compare the means of the baseline and post‐intervention results. McNemar chi‐square tests were used to determine the statistical significance of HbA1c values in relation to the 53 mmol/mol glycaemic goal for health before and after the intervention [9]. Statistical significance was assumed when *p* < 0.05.

## 3 Results

Two hundred and ten participants attended DESMOND in the primary care centre during 2018. Of these, 66 participants returned for the locally developed 6‐month follow‐up session. There were no statistically significant differences in characteristics (age, weight, BMI) between those who attended just the first session (*n* = 144) and those who attended both sessions (*n* = 66). Those who returned for the 6‐month follow‐up session had a higher mean HbA1c at baseline compared to those who attended DSME only (59 mmol/mol (7.5%) vs. 53.78 mmol/mol (7.1%), *p* = 0.046).

Of those who attended the Midwest DSME (*n =* 66), the mean age was 63 years (SD:11 years). The length of time since diagnosis ranged from 3 months to 11 years. Clinical characteristics are shown in Table 1. Mean baseline HbA1c was 58.4 mmol/mol (7.5%) (SD: 16 mmol/mol). Mean HbA1c at follow‐up was 52 mmol/mol (6.9%) (SD: 10.43 mmol/mol) (*p = 0.006*). At follow‐up, 60% of participants had a reduction in HbA1c, with values ranging from 1 to 47 mmol/mol. At baseline, 52% (*n* = 27) were below the 53 mmol/mol (7%) glycemic goal recommended for most individuals with type 2 diabetes mellitus. This increased to 71% (*n* = 37) at follow‐up (*p < 0.001*) (Table 2).

**TABLE 1:** Baseline and 6‐month clinical data of participants (*n* = 66) who completed primary care diabetes self‐management education.

	*N*	Mean	Range	Min.	Max.	Std. deviation
Age	66	63.17	37.00	43.80	80.80	10.73
Height	60	1.70	0.39	1.49	1.88	0.095
Baseline weight	63	93.39	97.80	55.00	152.80	20.01
Weight at 6 months	56	91.98	91.20	53.30	144.50	19.98
Difference in weight	54	−1.3833	25.20	−19.00	6.20	4.39
Baseline HbA1c (mmol/mol)	65	58.42	58.00	41.00	99.00	16.06
Baseline HbA1c (%)	65	7.5	7.5	5.9	11.2	3.6
Difference in HbA1c	52	−5.73	84.00	−47.00	37.00	15.02
HbA1c at 6 months (mmol/mol)	52	51.88	57.00	40.00	97.00	11.91
HbA1c at 6 months (%)	52	6.9	7.4	5.8	11	3.2

**TABLE 2:** Changes in Hb1Ac above/below 53 mmol/mol (7%) cut‐off [17] at baseline and 6‐month follow‐up.

HbA1c	Baseline (*n* = 52)	6 months (*n* = 52)
≤ 53 mmol/mol (7%)	27 (52%)	37 (71%)
> 53 mmol/mol (7%)	25 (48%)	15 (29%)

Mean bodyweight at baseline was 93.4 kg (SD: 20 kg). At follow‐up, mean body weight was 92 kg (SD: 19.98 kg). This mean decrease of 1.4 kg represented a 1.5% reduction in mean bodyweight (*p = 0.21*). A subset analysis of BMI found that the percentage of individuals in the normal weight category (BMI: 18.5–25 kg/m^2^) increased from 9.4% to 13.2% at follow‐up. This coincides with a corresponding decrease in the overweight BMI category (BMI: 25–29.9 kg/m^2^) from 30.2% to 26.4%. The percentage of those in the obese category (BMI > 30 kg/m^2^) remained unchanged at 60.4%. Although the number of participants in the obese category remained unchanged (*n* = 32), it was observed that 18 participants (56%) recorded a mean weight loss of 4.4 kg (range 0.8–11.4 kg). Of these 18 individuals, 39% (*n* = 7) lost > 5% body weight, a clinically significant marker [8].

## 4 Discussion

The aim of this study was to evaluate the efficacy of the DESMOND programme in participants who returned for a locally developed 6‐month follow‐up session delivered by registered dietitians and a diabetes nurse specialist on key clinical outcomes. Our results found that HbA1c was reduced by 6.54 mmol/mol (2.7%) at follow‐up. This is comparable to previous work conducted in the UK by Chatterjee et al. [20] who reported a 10 mmol/mol (3.1%) reduction in HbA1c at 6 months post‐intervention. Similar findings have been reported across diverse populations attending other similar DSME programmes including China [21] and Latino adults [22].

As the cohort's mean age was 63 years, 53 mmol/mol (7%) was selected as the glycaemic goal for this study. The ADA recommends an HbA1c target of 53–58 mmol/mol (7%–7.5%) in older adults (> 65 years) who are otherwise healthy with few existing comorbidities [9]. In practice, HbA1c goals are person‐centred and set in collaboration between the individual and healthcare professional [9]. Therefore, these results may potentially underestimate the benefits of DSME, as they do not account for individuals with more lenient glycaemic goals, such as older individuals, those with comorbidities, longer time since diagnosis, or those who cannot independently manage their condition.

The UK Prospective Diabetes Study (UKPDS) was one of the largest and longest studies ever undertaken in diabetes with a median follow‐up of 10 years, which found that there was no threshold in terms of clinical benefits of improving HbA1c values [23]. Hence, any improvement in glycaemic control was seen to be beneficial for health [23]. An epidemiological extrapolation of this study showed that each 1% reduction in mean HbA1c was associated with significant decreases in risk for any diabetes end‐point (diabetes‐related mortality, myocardial infarction and microvascular complications) with no threshold of risk being observed for any end point [23]. More recent research using data from the UK Biobank of 471,399 (56% women) individuals without cardiovascular disease established an 18% greater risk of a myocardial infarction in both sexes with each 1% higher HbA1c [24]. In this present study, 60% of participants saw a reduction in HbA1c and, therefore, the potential for future health benefits.

We found greater than 90% of participants were overweight/obese at baseline. This may be an indication of the close correlation between increased adiposity and type 2 diabetes mellitus risk [25]. According to the Model of Integrated Care published by the Irish Health Service Executive, weight management is one of many interventons that may hep to optimise glucose control in those with type 2 diabetes mellitus who are overweight/obese [11]. In this study, the mean decrease of 1.4 kg (1.5%) found at follow‐up, albeit not statistically significant, is comparable to previously published work [13]. A modest weight loss improves glycaemic control and reduces the need for glucose‐lowering medication [25]. A meta‐analysis conducted by Franz et al. [26] concluded that a > 5% weight reduction equated to a 7 mmol/mol HbA1c reduction in those with established type 2 diabetes mellitus and up to a 13 mmol/mol reduction in newly diagnosed individuals.

Our findings report that 13% of participants experienced weight loss of more than 5%. The ADA has reported that 3%–7% weight loss improves glycaemia in those with diabetes, while also referencing the importance of small attainable weight loss goals targets [25]. It is important to acknowledge that the mean age in this study was 63 years. Central obesity was the strongest characteristic associated with diabetes in older Irish adults [6]. Weight loss in older individuals with type 2 diabetes mellitus has been contra‐indicated by ESPEN as they acknowledge that following a weight‐reducing diet may result in loss of muscle mass and functional decline [27]. The prevalence of malnutrition in older people with diabetes is as high or even higher than matched individuals without diabetes [27]. If weight reduction is beneficial, it should be approached with great care, preferably in an individual session with a dietitian offering personalised support and not general DSME. It is also important to note that DESMOND is not a weight loss intervention, and participants are encouraged to choose their own risk factor for intervention. This could include physical activity, smoking, managing cardiovascular risk factors, or a psychosocial aspect. Participants, in collaboration with the educator, then set goals around their chosen risk factor, which may not have been HbA1c or weight‐related.

We noted a high rate of attrition with just 31% (*n* = 66) participants returning for the 6‐month follow‐up session. The motivation to attend the follow‐up session may be related to the observed higher HbA1c at baseline among those who attended [2, 3]. The role of HCPs in facilitating participation in DSME at critical stages of care on an ongoing basis among people with diabetes is highlighted to support ongoing self‐management and decision‐making [2, 3]. Since this study was conducted, the Covid‐19 pandemic has had an impact on healthcare globally and has resulted in huge delays in the delivery of DSME. While services are being restored, HCPs continue to face many challenges, including increased waiting lists, delayed diagnoses, or those who remain fearful of entering a healthcare setting environment [28]. Covid‐19 has also had an impact on health‐related behaviours such as physical activity and maintenance of a healthy weight, which may indirectly affect those with diabetes. The need for referral to DSME may be more important now than prior to the pandemic [28].

### 4.1 Strengths and Limitations

As this is an observational study, we cannot determine cause and effect. This study focused solely on clinical outcomes within the scope of the available data for reporting proposed DSME core outcome measures [3]. However, scant empirical evidence exists regarding the impact of interventions spearheaded by dietitians in real‐world contexts. Consequently, we deemed it imperative to contribute to this body of knowledge, particularly concerning the pragmatic aspects of implementing DSME within primary care settings. In practical terms, DSME educators depend on both electronic patient lab systems and patient self‐reports of clinical outcomes. However, it is noteworthy that lab results frequently lack recent (within 3 months) biochemical data, particularly when they have not been requested at the point of referral to DSME in primary care. Hence, any changes seen cannot be solely attributed to the DESMOND education. It also needs to be considered that changes may have come about due to the participants being highly motivated individuals and opting to attend DSME. It should also be noted that this study took place prior to the Covid‐19 pandemic and prior to the widespread introduction of telemedicine. During the pandemic, DSME moved to online platforms. DSME provided via telemedicine has previously been shown to improve glycemic control, diabetes knowledge, and self‐care adherence behaviour [29]. Future research should examine the effectiveness of telemedicine with regard to improving attendance at DSME sessions. An option for telemedicine may have improved attendance in this present study.

Despite the number of participants attending DESMOND initially, only 66 attended the locally developed follow‐up session and had repeat clinical data to be included in this study. Future research should focus on reasons for non‐attendance to DSME and in particular the HCP role in helping participants to understand the importance of DSME as part of treatment plans in integrated care models [11]. Due to demands on the local dietetic service, this study did not have the staffing resources available to collect additional measurements of body composition (e.g., waist circumference, waist‐to‐hip ratio); future research should also evaluate the changes in these anthropometric measurements alongside potential changes in HbA1c. Notably, the influence of gender on attendance rates and trajectory of type 2 diabetes mellitus cannot be ignored. Previous research conducted in this area has shown that in 365 individuals with newly diagnosed type 2 diabetes mellitus, attendance at structured education was independently associated with female gender (Odds Ratio (OR) 1.28, 95% CI 1.05–1.46), lower HbA1c (OR 0.98 mmol/mol 95% CI 0.97–0.99) and non‐smoker status (OR 1.36, 95% CI 1.07–1.55) [30]. Therefore, future research should examine ways to improve attendance for males.

Regardless, this is an evaluation of the effectiveness of the Midwest DSME impact on clinical markers in participants who completed the programme. In terms of strengths, this sample was representative of individuals in Ireland with type 2 diabetes mellitus. The DESMOND programme has been previously validated and ensures generalisability of these findings across DESMOND programmes.

## 5 Conclusion

In a sample cohort of adults with ongoing and newly diagnosed type 2 diabetes mellitus, completion of the DESMOND programme resulted in sustained benefits in HbA1c and BMI clinical outcomes at 6‐month follow‐up. The importance of DSME for type 2 diabetes mellitus is now recognised, and this is reflected in national and international policies [2, 11, 12]. As the rates of type 2 diabetes mellitus continue to rise, further research is required to determine optimal contact time and frequency of sessions required in order to sustain the observed improvement in clinical outcomes.

## Author Contributions

C.S.: formal analysis, methodology, writing – original draft, writing – review and editing. T.O.: conceptualization, data curation, formal analysis, methodology, writing – review and editing. A.G.: Conceptualization, formal analysis, methodology, writing – review and editing.

## Conflicts of Interest

The authors declare no conflicts of interest.

## Data Availability

The data that support the findings of this study are available from the corresponding author upon reasonable request.
